# Double low protocol in pediatric abdominal CT for evaluating right lower quadrant pain

**DOI:** 10.1007/s11604-025-01766-w

**Published:** 2025-03-29

**Authors:** Hyun Jeong Park, Hyewon Choi, Rae Rim Ryu

**Affiliations:** Department of Radiology, Chung-Ang University Hospitall, Chung-Ang University College of Medicine, Seoul, Republic of Korea

**Keywords:** Child, Contrast media, Tomography, X-ray computed, Abdomen, Acute, Radiation dosage

## Abstract

**Purpose:**

In pediatric patients, minimizing radiation and contrast media exposure without compromising diagnostic accuracy is paramount. Double low protocol, which utilizes a low dose contrast concentration and low tube voltage, could be a safer alternative. We compare diagnostic efficacy of double low protocol (Group A, 240 mgI/ml + 80 kVp) with conventional protocol (Group B, 350 mgI/ml + 120 kVp) in pediatric patients (< 10 years) presenting with abdominal pain and suspected acute appendicitis.

**Materials and methods:**

This retrospective study included 121 pediatric patients who underwent enhanced abdominal CT between January 2019 and February 2023: 62 with Group A and 59 with Group B. We compared radiation dose, iodine load, and quantitative image quality parameters. Two radiologists independently assessed diagnostic image quality on a 5-point scale, visualization of the appendix, and diagnostic performance for acute appendicitis and its complications.

**Results:**

There were no significant differences in mean age (7.6 ± 2.0 vs. 7.6 ± 2.1, *p *= 0.956), body weight (31.4 ± 11.2 kg vs. 31.7 ± 11.4 kg, *p* = 0.972), and contrast media volume used (59.3 ± 21.0 ml vs. 65.0 ± 20.0 ml, *p *= 135) between the two groups. However, effective dose and iodine load used were significantly lower in Group A compared to Group B (2.7 ± 1.1 mSv vs. 4.3 ± 1.5 mSv and vs. 12.7 ± 4.6gI vs.18.6 ± 6.7gI, all *p* < 0.001). Although diagnostic image quality, noise and signal-to-noise ratio were significantly lower in Group A, visualization of the appendix (*p* = 0.853) and diagnostic accuracy for appendicitis were comparable between the two groups (98.4% vs. 94.9%, *p* = 0.284).

**Discussion:**

The double low protocol offers an effective alternative for evaluating pediatric patients requiring enhanced abdomen CT, achieving comparable diagnostic performance while significantly reducing radiation dose. We believe that our findings support safer CT acquisition practices for pediatric patients requiring enhanced CT imaging.

## Introduction

As computed tomography (CT) plays a pivotal role in diagnosing various disease due to their high diagnostic accuracy, usage of CT in clinical practice is increasing. In this era, optimizing acquisition parameters to reduce radiation dose is crucial, particularly in pediatric patients. Radiation-induced carcinogenesis is widely accepted, and the pediatric patients are more susceptible to injury due to their rapid cell division [1]. Several strategies, including the use of low tube voltage and image noise reduction techniques such as iterative reconstruction (IR), deep learning-based noise reduction, are widely adopted to reduce radiation dose [2–4].

For safer enhanced CT acquisition, reducing the exposed dose of iodine is also beneficial. Acute adverse events from contrast media are categorized into allergic and chemo-toxic physiological reactions, with the latter being proportional to the injected iodine dose [5]. Contrast-induced nephrotoxicity and thyroid toxicity are the representative adverse chemo-toxic event. Accordingly, the Food and Drug Administration (FDA) recommends thyroid monitoring in pediatric patients younger than 3 years when exposed to the iodine contrast media for medical imaging [6].

Performing CT scans with low tube voltage and low iodine concentration contrast media, namely double low protocol, would be ideal in pediatric patients. However, the resultant decrease in image quality must be considered. Nevertheless, several studies have demonstrated the feasibility of double low protocol in pediatric patients [7–9]. Sun et al. [7] improved image quality of abdomen CT, acquired with 100kVp and low contrast dose (270mgI/ml) with use of IR. You et al. [8] showed comparable signal-to-noise (SNR), contrast-to-noise ratio (CNR) and diagnostic image quality in pediatric abdomen CT acquiring 70kVp with use of 250mgI/mL contrast media compared to 80–100kVp and 350mgI/mL contrast media. However, previous studies only compared the quantitative or qualitative image quality rather analyzed the impact on actual diagnostic accuracy.

Meanwhile, the appendix is a small tubular structure, necessitating appropriate image quality for accurate delineation. Moreover, CT is frequently employed in diagnosing acute appendicitis in children when ultrasound fails to provide a definitive diagnosis. In this study, our hypothesis is that the double low protocol does not impair diagnostic performance. Therefore, our aim in this study is to compare the image quality and diagnostic accuracy of enhanced abdomen CT in children presenting with abdominal pain and suspected acute appendicitis.

## Materials and methods

This retrospective study was approved by the institutional review boards and the requirement for informed consent was waived.

### Study population

**Fig. 1:**
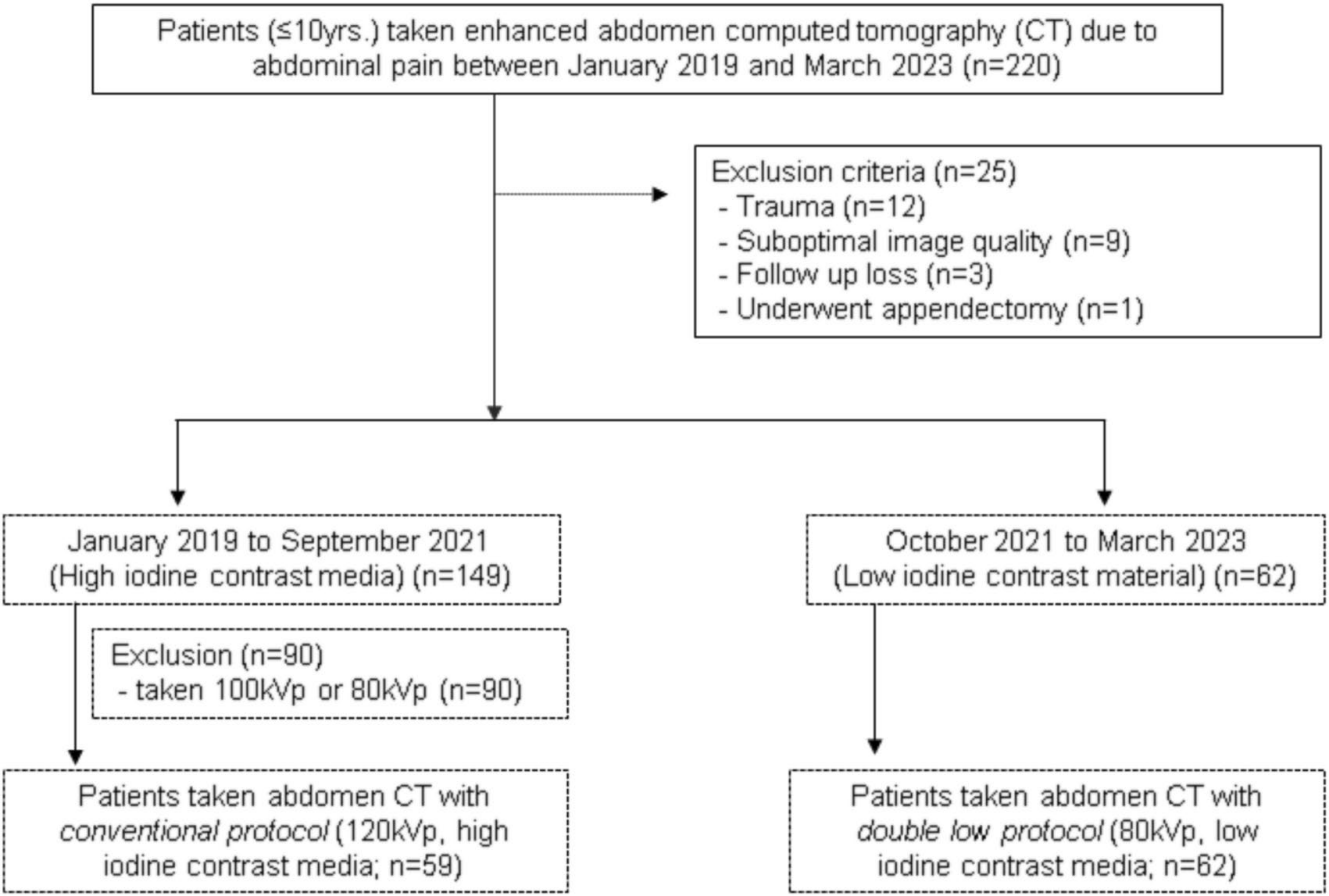
Flow diagram of patient inclusion and exclusion

Between January 2019 and February 2023, we conducted a retrospective review of clinical data and imaging findings from abdominal CT scans in pediatric patients under 10 years of age who visited the emergency room with complaints of abdominal pain. We excluded patients as follows: (a) other causes such as trauma, except for those presenting with spontaneous abdominal pain; (b) those with suboptimal image quality due to motion artifact; (c) those who underwent appendectomy; (d) patients with suspected appendicitis without surgical confirmation; and (e) patients with a non-surgical abdomen who were lost to follow-up. Until September 2021, our institution employed a conventional CT protocol for pediatrics, utilizing a tube voltage of 120 kVp along with various high concentration contrast media (350mgI/mL), including Iobrix® (Taejoon Pharmaceutical, Seoul, South Korea), Omnipaque® (Nycomed Amersham, Oslo, Norway), Iomeron® (Bracco, Milan, Italy), and Bonorex® (Central Medical Service, Seoul, South Korea). From October 2021, we have routinely implemented a double-low protocol, utilizing a low tube voltage of 80 kVp and low-concentration iodine contrast media (240 mgI/mL iohexol [Iobrix® inj. 240, Taejoon pharm Co. Ltd., Seoul, Korea]) in pediatric patients younger than 10 years. From this period, a low-concentration iodine contrast media (240 mgI/mL iohexol) was introduced at our institution. Because, several studies that evaluated the image quality of the double low protocol were published, and addressed their non-inferiority compared to conventional CT [7–9], we adopted the double low protocol as part of a policy to reduce both radiation dose and iodine exposure in pediatric patients. We categorized CT examinations into two groups based on the protocol used: Group A (double low protocol), and Group B (conventional protocol), including a total of 121 patients, with 62 in Group A (Fig. 1).

### CT protocol

Abdominal CT scan was performed with a multidetector CT scanners (Brilliance-iCT; Philips Healthcare, Cleveland, OH, USA; Brilliance 64; Philips Healthcare; IQon-Sepctral CT, Philips Healthcare; Optimal 660, GE Healthcare; Revolution frontier, GE healthcare). Contrast media injection (1.5–2.0 mL/kg, 2 mL/sec) was performed intravenously [10] followed by 20 mL of saline via a commercially available power injector (Stellant D, Medrad, Warrendale, PA, USA). The acquisition parameters were as follows; slice thickness 3 mm with a 3-mm interval; rotation time, 0.5 s; pitch, 1 (Philips Healthcare), and slice thickness 2.5 mm with a 2.5 mm interval; rotation time, 0.4 s; pitch, 0.9 s (GE healthcare); automatic tube current selection (CAREDose 4D, Siemens Healthineers) with reference mAs 130; collimation 128 × 0.6; automated tube voltage modulation (CARE kV, Siemens Healthineers) with reference kV 120 in group A and 80 in group B.

The scan range was from just above the diaphragm to the top of the iliac crests. Using the bolus tracking method, scan timing was determined 70 s after starting the contrast media injection. CT images were obtained in the craniocaudal direction. All CT images were reconstructed using IR algorithm (iDOSE 4 for Philips Healthcare vendors and ASIR 30 for GE healthcare vendors).

### Image analysis

#### Radiation dose and iodine load

We assessed radiation dose of two groups. CT dose index volumes (CTDIvol) and dose-length product (DLP) were obtained using picture archiving and communication system PACS (M-View, Marotech, Seoul, Korea) in which the values were automatically displayed in the dose page of each scan. The estimated effective dose (ED) was obtained by multiplying DLP by a conversion factor of 0.015 or 0.020 mSv/mGy/cm according to the patients’ age [11]. The total iodine load for each patient was determined using the following equation: body weight (kg) × 1.7 mL/kg × iodine concentration (mgI/mL; 240 for Group A, 350 for Group B) [8] We also investigated adverse events related to the contrast agent used in CT imaging.

#### Quantitative image analysis

CT attenuation (HU) measurements and the definition of image noise as the standard deviation (SD) of CT attenuation were conducted by an attending radiologist. Circular regions of interest (ROIs) were positioned in the liver, pancreas, renal cortex, aorta, and paraspinal muscle. In each patient, efforts were made to maintain a consistent size and shape for each ROI as much as possible. Measurements of liver parenchyma and paraspinal muscle were performed at the main portal vein level. For the liver, the attenuation was calculated as the mean of four ROIs obtained from the anterior and posterior segments of the right lobe, and the medial and lateral segments of the left lobe, with attention to avoiding areas of inhomogeneity and vessels. The attenuation of the paraspinal muscle was calculated as the mean of the ROIs measured on both sides, with care taken to avoid fat and blood vessels. ROIs were placed in the renal cortex while avoiding the medulla to evaluate the kidney. Contrast-to-noise ratios (CNRs) and signal-to-noise ratios (SNRs) of the liver, pancreas, renal cortex, and aorta were calculated using the following equations: CNR = (ROIo – ROIm)/SDn; ROIo = attenuation of the organ, ROIm = attenuation of the paraspinal muscle, SNR = ROIo/SDn [12].

#### Qualitative image analysis

For subjective analysis, two board-certified radiologists, blinded to the patients’ clinical information, independently reviewed the CT images using a commercial workstation equipped with a PACS monitor (2000 × 2000 pixels). The reviewers were allowed to vary the window width and level as they would in clinical practice. We evaluated the following aspects within the images: (1) diagnostic image quality, (2) visualization of the appendix, (3) overall likelihood of acute appendicitis, (4) presence of appendiceal perforation, and (5) final CT diagnosis. *Diagnostic image quality* was assessed using a 5-point scale: 5 for well-depicted with minimum or no noise, 4 for less than average noise with high confidence, 3 for average noise with acceptable image and moderate diagnostic confidence, 2 for above average noise with low confidence, and 1 for unacceptable noise. *Visualization of the appendix w*as evaluated on a 3-point scale: 2 for entirely visualized, 1 for partly visualized, and 0 for not visualized. *The overall likelihood of acute appendicitis* was rated on a 5-point scale: 5 for definitely acute appendicitis, 4 for probably acute appendicitis, 3 for indeterminate finding for appendicitis, 2 for probably not appendicitis, and 1 for definitely not appendicitis. *Presence of appendiceal perforation* was assessed on a 3-point scale: 2 for presence, 1 for indeterminate, and 0 for absence. *Final CT diagnosis* was categorized into 3 groups: no pain focus, non-surgical abdomen, and appendicitis. The diagnostic criteria for appendicitis on CT scan were defined as follows: a dilated appendix with a diameter greater than 6 mm, accompanied by mural thickening and fat infiltration in the mesoappendix, with or without the presence of an appendicolith, phlegmon, or abscess [13]. In cases where the appendix was only partly visualized, but the remaining portion showed inflammatory changes, we considered it indicative of appendicitis.

### Standard reference of final diagnosis

We reviewed patients’ hospital records, including surgical, pathological reports, and medical records. For cases diagnosed with acute appendicitis based on CT imaging, the final diagnosis was established through surgical and pathological findings. For patients who did not undergo surgery, the final diagnosis was determined by reviewing the patient’s medical records for the 6 months following their initial visit.

### Statistical analysis

The patient’s characteristics and CT-related factors, including radiation dose and the amount of iodine load, were compared between Groups A and B using Fisher’s exact tests for categorical variables and the Student’s *t* test for continuous variables. The Mann–Whitney U test was applied to compare qualitative image analysis scores between Groups A and B. Diagnostic accuracy, sensitivity, and specificity were calculated for both observers based on the assumption that a confidence level of 3 or higher was considered positive for the diagnosis of acute appendicitis. In cases of appendiceal perforation, a likelihood rating of 2 was considered positive. Cohen’s kappa statistics were employed to assess the degree of inter-reader agreement for qualitative analysis using MedCalc. The weighted kappa value was interpreted as follows: 0.81–1.00, excellent agreement; 0.61–0.80, substantial agreement; 0.41–0.60, moderate agreement; 0.21–0.40, fair agreement; < 0.20, poor agreement [14]. Lastly, we conducted logistic regression analysis to identify the factors that affect the diagnostic image quality and visualization of appendix. Multivariate regression analysis was performed using stepwise method. Data were analyzed using IBM SPSS Statistics for Windows (Version 21.0, IBM Corp., Armonk, NY). A *p* value of < 0.05 was considered statistically significant. Mean and standard deviation were calculated for quantitative data.

## Results

### Baseline patients’ characteristics

Patient characteristics and comparison between the two groups are summarized in Table 1. There was no significant difference in age, sex, weight, BMI, CT machine, and the volume of contrast media between the groups (all, *p* > 0.05). Among the 121 patients, 22 (18.2%) were diagnosed with acute appendicitis, with 9 (14.5%) in Group A and 13 (22%) in Group B. A total of 66 patients presented with a non-surgical abdomen, including nonspecific inflammation in gastrointestinal tract (*n* = 59), mesenteric lymphadenitis (*n* = 3), and genitourinary tract problem (*n* = 4). The remaining 55 patients who underwent CT examinations without pain focus showed clinical improvement during follow-up.

**Table 1:** Baseline characteristics of study participants

Variables	Total (*n* = 121)	Group A (double low protocol, *n* = 62)	Group B (conventional protocol, n = 59)	*p*
Age (years)	7.6 ± 2.0	7.6 ± 2.0 (range, 4–10)	7.6 ± 2.1 (range, 4–10)	0.956
Male/female	77/44			
Weight (kg)	31.5 ± 11.2	31.2 ± 11.2	31.7 ± 11.4	0.972
Body mass index (kg/m^2^)	18.6 ± 3.5	18.3 ± 3.5	18.8 ± 3.5	0.949
CT machine (n)				0.521
iCT 256	98	55 (88.7)	43 (72.9)	
IQon-Spectral CT	11	4 (6.5)	7 (11.9)	
Brilliance 64	2	1 (1.6)	1 (1.7)	
Optima 660	7		7 (11.9)	
Revolution frontier	3	2 (3.2)	1 (1.7)	
Contrast media (n)				N/A
Iobrix 240	62	62 (100)		
Iobrix 350	18		18 (30.5)	
Omnipaque 350	13		13 (22.0)	
Iomeron 350	24		24 (40.7)	
Bonorex 350	4		4 (6.8)	
Contrast volume (ml)	62.1 ± 20.6	59.3 ± 21.0	65.0 ± 20.0	0.135
Iodine load (gI)	15.6 ± 6.4	12.7 ± 4.6	18.6 ± 6.7	< 0.001
Radiation dose				
CTDIvol (mGy)	4.2 ± 1.8	3.3 ± 1.4	5.2 ± 1.7	< 0.001
DLP (mGycm)	204.0 ± 100.7	159.3 ± 72.5	250.9 ± 105.2	< 0.001
Effective dose (mSv)	3.1 ± 1.5	2.7 ± 1.1	4.3 ± 1.5	< 0.001
Adverse events*	3 (2.5)	1	2	N/A
Acute appendicitis (n)	22 (18.2)	9 (14.5)	13 (22)	0.438

Data in parenthesis represent percentage for categorical variable. N/A, no available. Continuous variables represent as mean ± standard deviation

*A comparison between two groups was not performed due to the small number of event

Within 60 min following the administration of intravenous contrast media, mild acute adverse events were observed in three patients: one in Group A and two in Group B. One patient (Group A) reported mid-chest discomfort, which was relieved by painkillers, while the others (Group B) exhibited self-limiting symptoms such as nausea, vomiting, and urticaria. There was no significant difference between the two groups in terms of adverse events.

### Radiation dose and iodine load between two groups

**Fig. 2:**
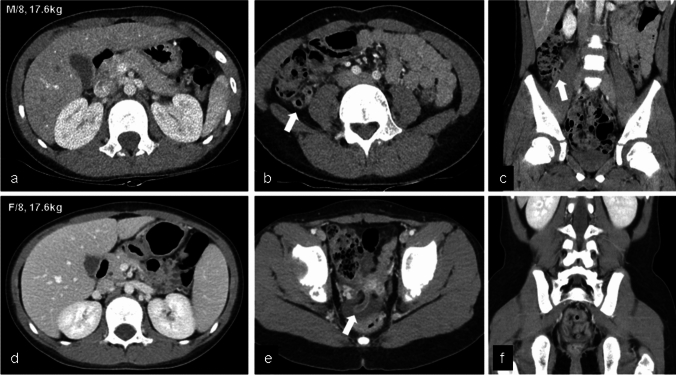
Double low protocol (**a**-**c**), and conventional protocol (**d**-**f**) in same age, injected contrast volume as 40 ml, and similar body weight. Appendix is fully well delineated in both protocols (arrows). The effective dose and iodine load used were 1.49 mSv and 10.9gI in the double low protocol group, and 3.97 mSv and 17.4gI in the conventional protocol group

For the assessment of radiation dose reduction, the mean CTDIvol, mean DLP, and mean ED were significantly lower in Group A compared to those of Group B (Table 1). Specifically, the mean CTDIvol, DLP, and ED of Group A were reduced by 36.6%, 36.5%, and 37.3%, respectively, compared to Group B. There was no significant difference in the volume of contrast media between the two groups (59.3 ± 21.0 mL in Group A and 65.0 ± 20.0 mL in Group B, *p* = 0.135). However, the mean iodine load of Group A was statistically significantly lower than that of Group B (*p* < 0.001). In comparison with Group B, the iodine load in Group A was decreased by 31.7% (Fig. 2).

### Quantitative image analysis

Quantitative analysis for CT parameters between both groups is shown in Table 2. Although the mean attenuation of liver, pancreas, renal cortex in Group A was higher than those in Group B, this difference did not reach statistical significance (*p* > 0.05). However, the attenuation of aorta was significantly higher in group A (*p* < 0.001). In terms of mean image noise across the organs, Group A showed significantly higher levels than those in Group B (all, *p* < 0.001). Although the CNR in Group A trended lower compared to that in Group B, a significant difference was observed only in the CNR of the renal cortex (*p* < 0.001). Furthermore, the SNR across three organs showed a significantly lower value in Group A compared to that in Group B (*p* < 0.001).

**Table 2:** Quantitative analysis of image quality

	Group A (double low protocol, *n* = 62)	Group B (conventional protocol, *n* = 59)	*p*
Liver			
Mean attenuation	132.6 ± 23.6	130.8 ± 27.8	0.698
Noise	13.7 ± 5.9	9.8 ± 2.4	< 0.001
CNR	5.9 ± 3.2	6.7 ± 2.7	0.170
SNR	11.6 ± 5.2	14.1 ± 4.4	0.004
Pancreas			
Mean attenuation	123.3 ± 19.9	119.0 ± 16.1	0.197
Noise	15.3 ± 6.8	9.8 ± 2.5	< 0.001
CNR	4.4 ± 2.6	5.1 ± 2.0	0.102
SNR	9.5 ± 4.1	12.8 ± 3.6	< 0.001
Renal cortex			
Mean attenuation	229.9 ± 45.1	215.5 ± 36.3	0.056
Noise	13.3 ± 5.9	9.4 ± 2.9	< 0.001
CNR	6.2 ± 2.1	9.4 ± 4.3	< 0.001
SNR	20.5 ± 9.4	25.1 ± 8.7	0.007
Aorta			
Mean attenuation	221.6 ± 45.0	195.1 ± 33.2	< 0.001
Noise	15.7 ± 8.5	11.6 ± 4.2	0.001
CNR	12.5 ± 7.6	12.0 ± 5.9	0.637
SNR	17.9 ± 9.9	18.9 ± 7.9	0.557
*SNR* signal-to-noise ratio, *CNR* contrast-to-noise ratio			

### Qualitative image analysis

**Fig. 3:**
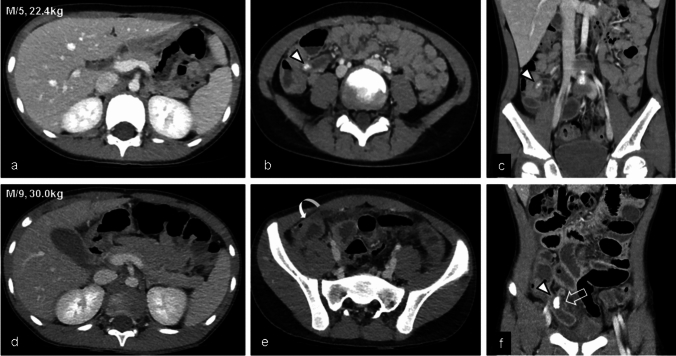
Correctly diagnosed acute appendicitis using the double low protocol without perforation (a-c), and with perforation (d-f). (**a**-**c**) A 5-year-old male patient weighing 22.4 kg. Entire appendix is well delineated as well as appendicolith at the ostium of appendix (arrowhead). (**d**-**f**) A 9-year-old male patient weighing 30 kg. Dilated appendix shows a large appendicolith (arrowhead) and focal wall defect at the medal portion of appendix, suggesting perforation (open arrow). Localized peritoneal thickening indicating peritonitis is also identified in the anterior portion of the right lower quadrant (curved arrow one)

Table 3 shows diagnostic performance of diagnosing acute appendicitis in both groups (Figs. 2 and 3). While the diagnostic image quality in both groups surpassed the acceptable level, characterized by average or lower noise levels, Group B showed superior quality compared to Group A (*p* = 0.004). In addition, there was a tendency for the lower age group (4–5 years) to exhibit a lower diagnostic image level; however, no significant difference was observed between Group A and Group B (*p* = 0.250). The majority of cases exhibited successful visualization of the appendix, with only three cases showing an invisible appendix (two in Group A, one in Group B). There was no significant difference between the groups for appendiceal visualization (*p* = 0.853).

**Table 3:** Diagnostic performance of acute appendicitis

	Group A (double low protocol, *n* = 62)	Group B (conventional protocol, *n* = 59)	*p*
Diagnostic image quality			0.004
3	7	2	
4	38	24	
5	17	33	
Mean diagnostic image quality according to age group			
4–5yrs	4 (*n* = 13)	4.2 (*n* = 12)	0.250
6–8yrs	4.2 (*n* = 23)	4.7 (*n* = 22)	0.013
9–10yrs	4.3 (*n* = 26)	4.5 (*n* = 25)	0.103
Visualization of appendix			0.853
Invisible	2	1	
Partial visible	11	10	
Cleary visible	49	48	
Final CT diagnosis			0.234
No pain focus	26	26	
Non-surgical abdomen	28	19	
Acute appendicitis	8	14	
Diagnostic performance of CT for diagnosing appendicitis			
*Sensitivity, %	88.9 (8/9)	92.3 (12/13)	0.789
*Specificity, %	100 (53/53)	95.7 (44/46)	0.129
*Accuracy, %	98.4 (61/62)	94.9 (56/59)	0.284
Cases of perforation	2	4	
Sensitivity, %	50	50	-
Specificity, %	85.7	100	0.169
Accuracy, %	77.8	84.6	0.691
Periappendiceal abscess^‡^	0/9	1/13	N/A

*Considered positive of appendicitis when likelihood rate ≥ 3

^†^Considered positive for perforation when likelihood rater = 2

‡A comparison between two groups was not performed due to the small number of event

The diagnostic performance for acute appendicitis showed similar results between the groups (*p* > 0.05). In Group A, the sensitivity, specificity, and accuracy were 88.9%, 100%, and 98.4%, respectively, while in Group B, they were 92.3%, 95.7%, and 94.9%, respectively. In three cases with appendiceal non-visualization, both radiologists correctly diagnosed the condition as less likely to be appendicitis.

The inter-reader agreement between two radiologists is presented in Table 4. Moderate to substantial weighted kappa values were observed for both the visualization of the appendix and appendiceal perforation in both groups, with no significant difference noted (*p* = 0.787 and 0.167, respectively). For the likelihood of acute appendicitis, Group A exhibited excellent inter-reader agreement compared to Group B (*k* = 0.932 and 0.708, respectively; *p* = 0.026).

**Table 4:** Inter-reader agreement of the likelihood of appendicitis

	Group A (double low protocol, *n* = 62)	Group B (conventional protocol, *n* = 59)	*p* value
Visualization of appendix	0.570 (0.353–0.757)	0.603 (0.358–0.848)	0.787
*Likelihood of appendicitis	0.932 (0.800–1.000)	0.708 (0.497–0.919)	0.026
^†^Perforation of appendicitis	0.659 (0.037–1.000)	0.658 (0.036–1.000)	0.167

*Considered positive of appendicitis when likelihood rate ≥ 3

^†^Considered positive for perforation when likelihood rater = 2

### Association between diagnostic qualitative image assessment and quantitative assessment

Table 5 presents the results of logistic regression analyses on factors influencing image diagnostic image quality (image diagnostic level 4–5 vs. 1-–) and visualization of appendix (clearly visualized vs. partially or not visualized). Good image diagnostic level was associated with quantitative image parameters such as liver CNR, live SNR, and aorta CNR on univariate analysis and liver CNR (OR, 1.60; 95% CI, 1.12–2.29, *p* = 0.01) remained independent factor on multivariate analysis. However, entirely visualization of appendix was associated with age (OR, 1.33; 95% CI, 1.06–1.67, *p* = 0.012).

**Table 5:** Association between qualitative diagnostic level and quantitative assessment

	Univariate analysis		Multivariate analysis	
	Odd ratio (95% CI)	*p* value	Odd ratio	*p* value
Diagnostic image quality				
Age	1.26 (0.91–1.76)	0.164		
Protocol, conventional	3.62 (0.72–18.23)	0.118		
Noise	0.91 (0.80–1.03)	0.168		
Liver CNR	1.60 (1.12–2.29)	0.002	1.60 (1.12–2.29)	0.010
Liver SNR	1.22 (1.03–1.44)	0.012		
Aorta CNR	1.28 (1.02–1.60)	0.009		
Aorta SNR	1.13 (0.98–1.29)	0.075		
Visualization of appendix				
Age	1.33 (1.06–1.67)	0.012	1.33 (1.06–1.67)	0.012
Protocol, conventional	1.16 (0.47–2.83)	0.749		
Noise	1.20 (1.02–1.41)	0.027		
Liver CNR	0.87 (0.75–1.00)	0.054		
Liver SNR	0.89 (0.82–0.98)	0.018		
Aorta CNR	0.96 (0.91–1.03)	0.271		
Aorta SNR	0.97 (0.93–1.01)	0.186		

*CNR* contrast-to-noise ratio, *SNR* signal-to-noise ratio

## Discussion

The principle of all radiation studies is ALARA (as low as reasonably achievable), emphasizing the importance of minimizing radiation exposure, particularly in pediatric patients. In this study, we reaffirmed the feasibility of double low protocol, which entails reducing both radiation dose and iodine exposure in pediatric enhanced abdominal CT scans. Although there were significant increases in image noise level (13.3–15.7 vs. 9.4–11.6, *p *< 0.001) and lower subjective diagnostic level (*p* = 0.004) in double low protocol compared to conventional protocol, both groups exceeded the acceptable diagnostic threshold. No significant differences were observed in the accuracy of diagnosing acute appendicitis (98.4% vs. 94.9%; *p* = 0.284) or in detecting perforation in cases of acute appendicitis (77.8% vs. 84.6%, *p* = 0.691) with substantial to excellent agreement (*k* = 0.932, and *k *= 0.659, respectively). Nevertheless, the double low protocol allowed for a reduction of 37.3% in the effective radiation dose and 31.7% in the iodine load.

To our knowledge, this is the first study that has proven the diagnostic accuracy of double low protocol in real clinical practices among pediatric patients. Although several parameters of quantitative and qualitative image quality were significantly lower in double low protocol, the diagnostic performance in present study was not compromised. Several studies have utilized a double low protocol for pediatric patients. You et al. [8] assessed the image quality using 70kVp and 250mgI/mL contrast media for abdominal CT in children. They reported the double low protocol was feasible while maintaining image quality, which reinforces our study results. In another study conducted in children with congenital heart disease [9], improved image quality was achieved despite the use of lower tube voltage and iodine load (70 kVp and 225 mgI/mL by diluting the 300 mgI/mL contrast media) due to the application of IR.

We demonstrated that subjective diagnostic image quality was influenced by quantitative parameters such as CNR and SNR. In our study, higher liver CNR was an independent factor for achieving satisfactory diagnostic image quality. However, entire visualization of appendix was significantly associated with patients’ age rather than qualitative image parameters, and possibly due to increased spatial resolution resulting from larger body size. In the present study, we employed the IR algorithm in all reconstructed CT scans across both groups. This approach enhanced image quality and reduced noise without compromising diagnostic accuracy.

Low tube voltage can significantly reduce the radiation dose. Theoretically, decreasing the tube voltage from 120 to 80 kvP can reduce radiation dose by up to 65%, due to the radiation’s proportional relationship to the square of tube voltage [15]. In our study, we achieved a radiation dose reduction of approximately 29.4%, aligning with previous study [8]. Another study reported up to 48% radiation dose reduction with double low protocol in adult chest CT scans [16]. Lower tube voltage can provide adequate image quality for smaller scanning object, owing to sufficient photon penetration [17]. Sun et al. [18] identified optimal tube voltage as 80kVp for children weighing under the 28 kg, and higher tube voltage is recommended for children weighting over 28 kg. In our study, the mean body weight of study population was 31.5 kg ± 11.2. With advancement of noise reduction technology, there is a need to expand the population that can benefit from low tube voltage. Likewise, CT acquisition with low tube voltage has also been attempted in adults in various organ [16, 19, 20]. Although some studies have reduced the tube voltage to 70kVp, we did not adopt it due to higher body weight of our study population and lower iodine concentration contrast media. In addition, we observed an increase in organ attenuation, particularly in aorta (226.1 ± 45.0 vs. 195.1 ± 33.2, *p* < 0.001) at 80kVp, despite using a lower concentration of iodine contrast media. This result can be attributed to the photoelectronic effect [17], and the phenomenon could be advantageous for detecting hyper-vascular lesions in abdomen CT in 80 kVp.

With the use of 240 mgI/mL contrast media, we were able to reduce the iodine load by 36.8%. To our knowledge, this is the first study that has proven the diagnostic accuracy of double low protocol in real clinical practices among pediatric patients. Therefore, we based the dose of contrast agent and radiation dose on previous studies [7–9], even though they are not identical to our study. Although our study demonstrated comparable results in terms of image quality in clinical practice when compared with previous studies, the potential reduction in side effects with a lower dose of contrast media remains unknown. This is because the relevant reference studies did not assess the relationship between the dose of contrast agent and side effects. Meanwhile, the FDA recently recommended routine thyroid function monitoring in infants and younger children within the 3 weeks’ exposure to iodinated contrast media [6]. Although there is debate, given that contrast-induced hypothyroidism is uncommon and most cases are transient, not requiring the treatment [21, 22], a single injection of iodine contrast media results in more than 1000-fold increase in iodine than the recommended daily intake [23]. Therefore, reducing iodine exposure as much as possible seems benefit. Regarding the contrast-induced nephrotoxicity, contrast media with higher viscosity lead to increased urine viscosity, making the renal tubules more susceptible to leading to injury [24], and the viscosity is strongly dependent on the iodine concentration [25]. Thus, the double low protocol could be beneficial in preventing chemo-toxic reaction from iodine contrast media. Meanwhile, hypersensitivity reaction is believed independent to iodine concentration and dose. In our study, a few patients exhibited minor hypersensitivity reaction to contrast media in both groups.

There are several limitations in our study. First, this is the single-centered, retrospective study with small sample size. A small portion of the patients presenting with abdominal pain were diagnosed with acute appendicitis, and among these, complicated cases, either perforated or formation of peri-appendiceal abscess, were even fewer. Second, due to the retrospective study design, various CT machines were used, and image quality could potentially be improved when taken with higher channel of CT machine. However, there was no difference in CT machines between the two groups, and most patients were scanned with the same CT machine, and use of the iterative reconstruction. However, we cannot rule out the possibility that image quality may influenced by unassessed factors such as radiographers’ level of expertise and patients’ level of cooperation. Fourth, the age of the patients included in this study ranged from 4 to 10 years, and no patients younger than 4 years included. Although the protocol did not impose an age restriction, it is likely that younger patients were primarily evaluated using ultrasound. Lastly, we focused on the diagnostic accuracy for diagnosing acute appendicitis among the patients presented with abdominal pain. In real clinical practice, enhanced CT is indicated for a wide range of conditions, necessitating additional study to determine the applicability of double low protocol for various clinical indication.

In conclusion, the double low protocol offers an effective alternative for evaluating pediatric patients requiring enhanced abdomen CT, achieving comparable diagnostic performance while significantly reducing radiation dose. We believe that our findings support safer CT acquisition practices for pediatric patients requiring enhanced CT imaging.

## Abbreviations

CNR: Contrast-to-noise ratio
CT: Computed tomography
CTDIvol: CT dose index volume
DLP: Dose-length product
ED: Effective dose
IR: Iterative reconstruction
ROI: Region of interest
SD: Standard deviation
SNR: Signal-to-noise ratio
